# Knowledge, Attitudes, and Practices Regarding Diabetes Mellitus Among Schoolteachers in Jordan: A Cross-Sectional Study

**DOI:** 10.3390/healthcare14111484

**Published:** 2026-05-27

**Authors:** Randa S. Alqaisi, Walid Al-Qerem, Basil Husam Al Tah, Eba’a Ibraheem Alhomedy, Belal Alzubi, Abdelhadi Alzaben, Ala’a Al-Dala’ien

**Affiliations:** 1 Department of Pediatric Endocrinology, Faculty of Medicine, Mutah University, Al-Karak 61710, Jordan; randasq@mutah.edu.jo; 2 Department of Pharmacy, Faculty of Pharmacy, Al-Zaytoonah University of Jordan, Amman 11733, Jordan; 3 Neurosurgery Department, Ibn-Alhytham Hospital, Amman 11194, Jordan; b.altah@ihh.com.jo; 4 Department of Pediatrics, Ministry of Health, Amman 11118, Jordan; ebaaibraeem@gmail.com; 5 Department of Special Surgery, Faculty of Medicine Mutah University, Al-Karak 61710, Jordan; belal88@mutah.edu.jo; 6 Department of Pediatrics, Faculty of Medicine, Mutah University, Al-Karak 61710, Jordan; dr_zaben@yahoo.com (A.A.);

**Keywords:** type 1 diabetes mellitus, schoolteachers, school health, diabetes management, emergency preparedness, school-based diabetes care, teacher training, knowledge, attitudes, and practices

## Abstract

**Background/Objectives:** Teachers are often the first adults responsible for supporting students with type 1 diabetes mellitus (T1DM) during the school day. This study assessed schoolteachers’ diabetes-related knowledge, attitudes, emergency readiness, and routine school practices in Jordan. **Methods:** A cross-sectional online survey was conducted among teachers working in public and private schools in Jordan between February and April 2026. The final sample included 604 teachers. Multivariable relationships were examined using path analysis in R with full information maximum likelihood. **Results:** The median age was 43 years (IQR: 38–47), 88.6% of participants were women, and 15.9% had received prior diabetes-related training. Median composite scores were 11/14 (IQR: 9–12) for knowledge, 43/50 (IQR: 39–46) for attitudes, 23/35 (IQR: 18–28) for emergency readiness, and 24/30 (IQR: 20–26) for routine practices. The path model showed close fit indices (chi-square(1) = 0.243, *p* = 0.622; CFI = 1.00; RMSEA = 0.00; SRMR = 0.001), although these indices were interpreted cautiously because the model had only one degree of freedom. Greater knowledge was associated with more favorable attitudes (beta = 0.374, *p* < 0.001), and more favorable attitudes were associated with better emergency readiness (beta = 0.141, *p* < 0.001) and routine practices (beta = 0.244, *p* < 0.001). Teachers with prior diabetes-related training also reported higher attitudes, emergency readiness, and routine practice scores. **Conclusions:** Jordanian teachers appeared willing to support students with diabetes, but this willingness was not matched by consistent emergency preparedness. Targeted training and stronger school-level protocols are needed to improve the safety and quality of diabetes care in schools.

## 1. Introduction

Diabetes mellitus remains one of the most pressing noncommunicable disease challenges worldwide [1], with particularly rapid growth in low- and middle-income settings. In the Middle East, the burden is substantial, affecting health systems, families, and social institutions, including schools [2,3,4].

For children and adolescents living with type 1 diabetes mellitus (T1DM), management extends well beyond the clinic. Much of their daily care takes place at school, where students may need blood glucose monitoring, timely access to food, adjustments during physical activity, and prompt support during episodes of hypo- or hyperglycemia. In Jordan, childhood T1DM is associated with a substantial treatment burden and can negatively affect daily functioning and quality of life [5,6].

The school environment is therefore central to safe diabetes management. Both the American Diabetes Association and the International Society for Pediatric and Adolescent Diabetes emphasize that students with diabetes should be supported in settings that can accommodate routine care and respond safely to acute events [7,8].

Although recent work in Jordan has started to address teachers’ preparedness to support students with type 1 diabetes in schools [9], the evidence base remains narrow. In particular, there is still limited national-level evidence spanning different school sectors and capturing teachers’ knowledge and attitudes, as well as their emergency readiness and routine day-to-day school practices. A clearer understanding of these domains is needed to identify training needs, inform school health policy, and improve both the safety and educational participation of students living with diabetes. Accordingly, this study aimed to assess schoolteachers’ knowledge, attitudes, and practices regarding diabetes and the management of students with diabetes in school settings across Jordan.

## 2. Materials and Methods

### 2.1. Study Design and Setting

A cross-sectional study was conducted among schoolteachers in Jordan to assess knowledge, attitudes, and practices related to diabetes mellitus and the management of students with diabetes in school settings. The survey targeted teachers working in public and private schools across the northern, central, and southern regions of the country. Data were collected online between February and April 2026.

### 2.2. Participants and Sampling

Eligible participants were schoolteachers who were actively teaching during the study period and agreed to participate. Teachers who declined participation or submitted unusable questionnaires were excluded from the relevant analyses. Recruitment used a nonprobability convenience sampling approach, and the questionnaire was distributed electronically through school administrations, teacher communication channels, and professional networks. This strategy was intended to broaden coverage across major regions and school systems.

The minimum required sample size was estimated using the single-population proportion formula, assuming a 95% confidence level, a 5% margin of error, and an expected proportion of 50% in the absence of prior national estimates. This yielded a minimum target of 384 participants [10]. A larger sample was sought to improve precision and reduce the impact of nonresponse.

### 2.3. Instrument Development

Data was collected using a structured, self-administered questionnaire developed after reviewing published instruments on teachers’ diabetes-related knowledge and school preparedness [11,12]. The final questionnaire was administered electronically through Google Forms.

Section 1 included 15 sociodemographic and professional items: age, sex, marital status, educational level, professional role, school type and sector, grade level taught, years of experience, region, availability of a school nurse, prior diabetes-related training, presence of students with diabetes in the classroom, respondents’ own diabetes status, family history of diabetes, and main sources of information. Section 2 assessed diabetes-related knowledge using 14 statements focused primarily on T1DM. Section 3 assessed attitudes toward students with diabetes using 10 Likert-type items. Section 4 assessed school practices using 13 Likert-type items, divided into emergency readiness and routine school practices.

### 2.4. Scoring System

The knowledge score was calculated from the 14 knowledge items in the questionnaire, coded as 1 for a correct response and 0 for all other responses, yielding a possible score range of 0 to 14. The attitude score was calculated as the sum of 10 Likert-type items (range 10–50), emergency readiness as the sum of 7 items (range 7–35), and routine practices as the sum of 6 items (range 6–30). Higher scores indicated better knowledge, more favorable attitudes, greater emergency readiness, and more supportive routine practices. Negatively worded attitude items were reverse coded so that higher values consistently reflected more positive attitudes. Composite scores were prorated when at least 50% of items within the relevant scale had been completed, in line with the supplied analysis dataset.

### 2.5. Validity and Reliability

Content validity was assessed by specialists in endocrinology, who reviewed the questionnaire for clarity, relevance, and appropriateness. A pilot test was conducted with teachers who were not included in the final analysis, and minor wording revisions were made before full deployment. Internal consistency of the final scales ranged from acceptable to excellent. Ordinal Cronbach’s alpha was 0.878 for knowledge, 0.880 for attitudes, 0.924 for emergency readiness, and 0.841 for routine practices.

### 2.6. Statistical Analysis

All statistical analysis we conducted using R version 4.5.3. Descriptive statistics were used to summarize participants’ characteristics and study variables. Categorical variables were reported as frequencies and percentages, whereas continuous variables and composite scores were summarized using medians and interquartile ranges (IQRs). Percentages were calculated based on the number of available responses for each variable.

**Figure 1:**
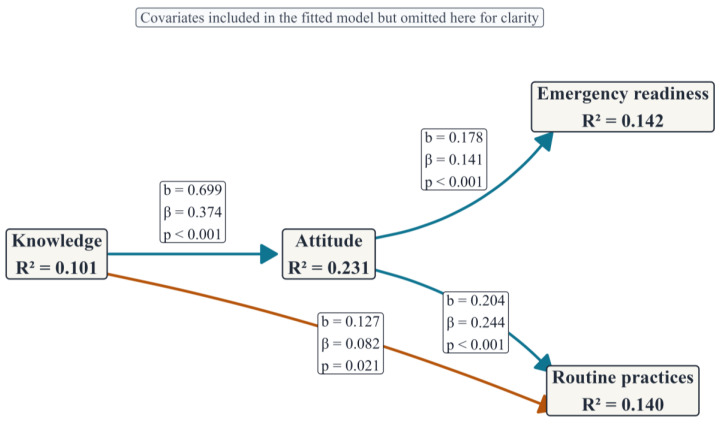
Hypothesized path model and final core direct effects from the fitted structural model. Note: Covariates were included in model estimation, but are omitted from the diagram for readability. Arrow labels show the unstandardized coefficient (b), standardized coefficient (beta), and *p*-value. R^2^ values are displayed inside the outcome boxes.

The path model was specified as a theory-informed extension of the knowledge-attitude-practice framework. In this framework, diabetes-related knowledge was treated as the most proximal informational domain because accurate understanding of type 1 diabetes is expected to shape teachers’ beliefs, perceived responsibility, and confidence in supporting affected students. Attitudes were then modeled as antecedents of emergency readiness and routine practices because these outcomes reflect teachers’ reported preparedness and school-support behaviours. Thus, the directional paths represent a prespecified conceptual ordering rather than evidence of temporal or causal effects. The hypothesized model, with the fitted core effects, is shown in Figure 1.

The main multivariable analysis used path analysis in R with the lavaan package. Knowledge, attitudes, emergency readiness, and routine practices were treated as continuous observed variables. The structural model specified that knowledge was regressed on demographic and school-related covariates; attitudes were regressed on knowledge and the same covariates; emergency readiness was regressed on attitudes and covariates, with the direct path from knowledge to emergency readiness fixed to zero; and routine practices were regressed on both knowledge and attitudes, in addition to the covariates. The adjusted path model controlled for age, years of teaching experience, sex, marital status, educational level, professional title, school type, school sector, grade level taught, school nurse availability, prior diabetes-related training, presence of students with diabetes in the classroom, respondent diabetes status, and family history of diabetes.

Missing data were handled using full information maximum likelihood. Because FIML relies on the assumption that missingness is ignorable conditional on observed variables, the extent of missingness was summarized and the final model was refitted using complete cases as a sensitivity analysis. Robust standard errors for the main model were estimated using the MLR estimator, whereas indirect and total effects were quantified using nonparametric bootstrap confidence intervals under maximum likelihood. Multicollinearity was assessed using variance inflation factors, with a maximum VIF below 4, indicating no major collinearity concerns. Because age and years of experience reflect closely related aspects of professional seniority, sensitivity analyses were conducted using alternative model specifications that retained only age or only years of experience.

### 2.7. Ethical Considerations

The study was conducted in accordance with the Declaration of Helsinki. Ethical approval was obtained from the Institutional Review Board of Mutah University before data collection. Participation was voluntary, informed consent was obtained from all respondents, and the questionnaire was completed anonymously. No names or other direct personal identifiers were collected, and online self-administration was used to allow teachers to complete the questionnaire privately without direct interviewer or school-administrator presence [13,14,15]. Data were stored securely and were accessible only to the research team.

## 3. Results

### 3.1. Participant Characteristics

Table 1 summarizes the characteristics of the 604 teachers included in the study. The median age was 43 years (IQR: 38–47), and the median teaching experience was 16 years (IQR: 9–22). Most respondents were women (n = 535, 88.6%), married (n = 512, 85.2%), and employed in public schools (n = 485, 80.3%). Mixed schools were the most commonly reported school type (n = 370, 61.5%), and elementary grades were the level most frequently taught (n = 323, 54.5%). Only 74 teachers (12.3%) reported that a school nurse was available at their workplace, whereas 96 (15.9%) had previously received diabetes-related training. In addition, 184 teachers (30.4%) reported having students with diabetes in their classroom, 375 (62.2%) had a first-degree relative with diabetes, and 42 (6.9%) reported having diabetes themselves. Median composite scores were 11 (IQR: 9–12) for knowledge, 43 (IQR: 39–46) for attitudes, 23 (IQR: 18–28) for emergency readiness, and 24 (IQR: 20–26) for routine practices.

### 3.2. Knowledge Regarding Type 1 Diabetes

Teachers’ knowledge regarding type 1 diabetes is presented in Table 2. Most teachers correctly recognized that type 1 diabetes is a chronic condition requiring ongoing management (85.8%) and that it results from insufficient insulin production (78.7%). In addition, 84.8% recognized the importance of regular blood glucose monitoring, and 81.2% were aware of potential complications such as kidney disease, nerve damage, and vision problems. Most participants also correctly agreed that children with diabetes can attend school as usual (86.0%) and participate in physical activity (59.0%). A large proportion correctly identified hypoglycemia symptoms such as shaking and sweating (79.2%), and 64.5% recognized insulin therapy as the main treatment. However, important gaps remained. Nearly two thirds (63.0%) incorrectly believed that excessive sugar consumption and lack of exercise are the main causes of type 1 diabetes, and only 49.1% correctly recognized that insulin does not cause dependency, indicating persistent uncertainty in some areas.

### 3.3. Attitudes Towards Students with Type 1 Diabetes

Table 3 presents teachers’ attitudes toward students with diabetes. Overall, teachers reported generally positive attitudes. More than one third strongly agreed that they were willing to work with students with diabetes in their classrooms (37.1%), and 43.4% strongly agreed that they were interested in attending diabetes management training programs. In addition, 42.9% strongly agreed that students with diabetes should be treated like other students, and 57.6% strongly agreed that teachers should receive training on managing diabetic emergencies.

Most teachers also supported the presence of a school nurse (69.6%) and the availability of glucometers in schools (68.2%). More than half (51.5%) strongly agreed that educational programs about diabetes should be mandatory. However, some less favorable views remained. Only 19.2% strongly disagreed with the statement that students with diabetes are a burden on schools and teachers, suggesting that negative perceptions persist among a proportion of respondents.

### 3.4. Emergency Readiness

As shown in Table 4, the most common response across most items was “agree”. Approximately 32.1% of teachers agreed that they were confident in recognizing symptoms of hypoglycemia, and 34.8% agreed that they knew the appropriate first response when a student with diabetes felt weak. Confidence was lower for more serious situations. Only 26.9% agreed that they knew the appropriate actions to take if a student with diabetes became unconscious, and just 22.0% strongly agreed that they knew how to administer glucagon. In addition, only 14.8% strongly agreed that their school had designated staff trained to manage diabetic emergencies, and only 20.6% strongly agreed that their school had a written emergency protocol for students with diabetes.

### 3.5. Routine School Practices

Routine school practices related to diabetes management are presented in Table 5. Most teachers agreed that students were allowed to monitor their blood glucose or eat snacks during class when needed (38.7%), and 48.9% agreed that teachers should communicate regularly with parents or school nurses about the needs of students with diabetes. However, fewer respondents reported supportive school-level practices. Only 25.8% indicated that their school provided appropriate food options for students with diabetes, and only 26.8% reported that physical activities were adjusted when necessary. In addition, 33.3% reported that their school organized educational or awareness sessions about diabetes.

### 3.6. Path Analysis

Figure 1 presents the hypothesized path model with the fitted core coefficients, and Table 6 and Table 7 report the corresponding direct, indirect, and total effects with standard errors and 95% confidence intervals. All direct, indirect, and total effects reported from this model are therefore covariate-adjusted estimates. Missingness was limited; no model variable exceeded approximately 7% missing data, and missingness for the four score variables ranged from 0.8% to 1.8%. Complete-case refitting gave the same direction and statistical significance for the core paths, with standardized estimates differing from the FIML model by no more than 0.018.

The retained path model showed close fit indices (chi-square(1) = 0.243, *p* = 0.622, CFI = 1.00, RMSEA = 0.00, and SRMR = 0.001). Because the retained model had only one degree of freedom, these global fit indices were interpreted cautiously. Adjusted direct effects for all predictors are presented in Table 6. Knowledge showed a strong positive association with Attitude (b = 0.699, 95% CI 0.494 to 0.904; β = 0.374; *p* < 0.001). Attitude was positively associated with both Emergency readiness (b = 0.178, 95% CI 0.070 to 0.290; β = 0.141; *p* < 0.001) and Routine practices (b = 0.204, 95% CI 0.143 to 0.282; β = 0.244; *p* < 0.001). After accounting for Attitude and covariates, Knowledge retained a smaller but statistically significant direct association with Routine practices (b = 0.127, 95% CI 0.018 to 0.232; β = 0.082; *p* = 0.021). Several covariates also demonstrated meaningful associations. For Knowledge, older age was associated with slightly higher scores (b = 0.047, 95% CI 0.004 to 0.097; *p* = 0.046), whereas more years of experience were associated with slightly lower Knowledge scores (b = −0.046, 95% CI −0.089 to −0.003; *p* = 0.030). Postgraduate education (vs. bachelor’s) was associated with lower Knowledge scores (b = −0.826, 95% CI −1.645 to −0.134; *p* = 0.027), and reporting having students with diabetes (vs. none) was associated with higher Knowledge (b = 0.967, 95% CI 0.541 to 1.417; *p* < 0.001). For Attitude, having received diabetes-related training was associated with higher Attitude scores (b = 1.195, 95% CI 0.182 to 2.139; *p* = 0.011), and personal diabetes was also associated with higher Attitude (b = 1.979, 95% CI 0.539 to 3.395; *p* = 0.006). For Emergency readiness, male sex (vs. female) was associated with higher readiness (b = 3.008, 95% CI 0.042 to 6.134; *p* = 0.040), being a manager (vs. teacher) was associated with higher readiness (b = 2.106, 95% CI 0.723 to 3.865; *p* = 0.005), nurse availability (yes vs. no) was strongly associated with higher readiness (b = 3.042, 95% CI 1.755 to 4.578; *p* < 0.001), and prior diabetes training was similarly associated with higher readiness (b = 3.268, 95% CI 1.825 to 4.728; *p* < 0.001). For Routine practices, diabetes training remained positively associated (b = 1.294, 95% CI 0.192 to 2.383; *p* = 0.008), and a first-degree family history of diabetes (yes vs. no) was associated with higher practice scores (b = 0.842, 95% CI 0.025 to 1.546; *p* = 0.023).

Indirect and total effects are presented in Table 7. Consistent with the constrained model specification, in which the direct path from knowledge to emergency readiness was fixed to zero, knowledge was associated with emergency readiness entirely through attitudes (indirect effect: b = 0.125, 95% CI [0.044, 0.203], β = 0.053, *p* = 0.002). For routine practices, knowledge showed a statistically significant total effect (b = 0.269, 95% CI [0.162, 0.372], β = 0.173, *p* < 0.001), consisting of both an indirect effect through attitudes (b = 0.142, 95% CI [0.088, 0.201], β = 0.091, *p* < 0.001) and a smaller direct effect (b = 0.127, 95% CI [0.018, 0.232], β = 0.082, *p* = 0.021), indicating partial mediation. Sensitivity analyses that alternately retained only age or only years of experience led to virtually identical conclusions. Across these alternative model specifications, the standardized core path coefficients changed by no more than 0.006, and the R^2^ values for the four outcomes changed by less than 0.008, suggesting that the main findings were not materially affected by how these two related covariates were modeled. To reassess model specification, a less constrained alternative model that additionally estimated a direct path from knowledge to emergency readiness was examined. This path was small and not statistically significant (b = 0.050, 95% CI −0.144 to 0.245; β = 0.021; *p* = 0.612), and the more parsimonious model with this path fixed to zero was retained.

Complete-case refitting was also performed to examine whether FIML handling of missing data materially influenced the model estimates. The complete-case model included 519 participants. The direction and statistical significance of the core paths were unchanged, and standardized estimates were very similar to the FIML estimates (knowledge to attitude: 0.374 vs. 0.385; attitude to emergency readiness: 0.141 vs. 0.125; attitude to routine practices: 0.244 vs. 0.226; knowledge to routine practices: 0.082 vs. 0.090). The indirect and total effects also led to the same substantive conclusions. Missingness frequencies for the model variables are shown in Appendix A, Table A1.

## 4. Discussion

This study provides broader national evidence on schoolteachers’ diabetes-related knowledge, attitudes, emergency readiness, and routine school practices in Jordan. Overall, teachers showed generally positive attitudes toward supporting students with diabetes, and many demonstrated a reasonable level of basic knowledge, particularly regarding the chronic nature of type 1 diabetes, the importance of blood glucose monitoring, and common symptoms of hypoglycemia. However, these strengths were accompanied by substantial gaps in emergency preparedness and inconsistency in school-level support. The findings suggest that willingness to support students with diabetes is present, but that willingness is not yet matched by the confidence, training, and institutional systems needed for safe care during the school day. Therefore, the relatively high attitude scores, despite only 15.9% of teachers reporting prior diabetes-related training, should be interpreted as evidence of willingness and perceived professional responsibility rather than adequate preparedness. This interpretation is consistent with studies showing that favorable teacher attitudes can coexist with limited school preparedness, low perceived competence, or weaker practice indicators [16,17,18]. In this sense, the low level of previous training does not contradict the attitude findings; rather, it suggests that diabetes training may be well received and could build on teachers’ existing willingness while improving practical knowledge, confidence, and emergency management skills [9,13,19].

This distinction between willingness and preparedness was most apparent in the emergency readiness domain. Teachers in this study were broadly supportive of students with diabetes, strongly endorsed the need for training, and expressed support for school nurses and glucometers. However, confidence was notably lower for high-risk situations, particularly responding to an unconscious student or administering glucagon. This pattern is consistent with previous work showing that teachers are often sympathetic and willing to help but feel underprepared when practical or high-risk management tasks are involved. In Spain, San Laureano et al. [16] found that teachers perceived their schools as inadequately prepared to manage diabetic emergencies and reported very limited specific training. Similarly, Wright et al. [17] reported limited diabetes knowledge and low perceived self-competence among school personnel in Georgia public schools.

The present findings also align with recent studies from the Middle East. In Saudi Arabia, it was reported that schoolteachers generally held positive attitudes toward supporting students with diabetes, but practical knowledge and readiness were uneven [11]. Likewise, Aljefree et al. [18] found moderate knowledge, favorable attitudes, and poor practice scores among public-school teachers in Jeddah. That combination is similar to the pattern seen in the present study, where attitudes were comparatively positive but routine practices and emergency readiness were less robust. Overall, these studies suggest that positive intentions alone do not ensure effective school-based diabetes care.

The knowledge findings in the current study show a similar mix of strengths and gaps. Most respondents correctly recognized that type 1 diabetes is a chronic condition requiring ongoing management and understood the importance of blood glucose monitoring. However, many still incorrectly attributed type 1 diabetes to excessive sugar intake and lack of exercise, and only about half correctly recognized that insulin does not cause dependency. Comparable misconceptions have been reported elsewhere. Alshammari and Haridi [20] in a study of public female elementary schools in northern Saudi Arabia, found that although teachers demonstrated a fair overall knowledge score, only 27.3% achieved a level considered good, and the authors concluded that teachers particularly needed stronger knowledge around recognizing and managing diabetic emergencies. Stefanowicz-Bielska et al. [12] likewise concluded that teachers’ knowledge was insufficient to ensure a safe school experience for a child with type 1 diabetes.

These misconceptions may have practical consequences, as misunderstandings about type 1 diabetes could affect how teachers interpret students’ needs and how confident they feel in providing support. In this study, greater knowledge was associated with more favorable attitudes, which in turn were associated with better emergency readiness and routine practices. This pattern aligns with qualitative findings from Horvath et al. [12] showed that teachers’ attitudes toward students with type 1 diabetes are closely tied to their knowledge, emotions, and beliefs about diabetes management, including misunderstandings about treatment. Together, these findings suggest that strengthening teachers’ understanding of type 1 diabetes may improve not only what they know, but also how prepared and confident they feel in supporting students at school.

The use of path analysis allowed this study to extend beyond descriptive findings and examine the interrelationships among knowledge, attitudes, emergency readiness, and routine practices. Greater knowledge was associated with more favorable attitudes, and more favorable attitudes were associated with both better emergency readiness and more supportive routine practices. In addition, knowledge had a significant total effect on routine practices, with part of that effect operating indirectly through attitudes. This suggests that knowledge may not influence school support only in a direct, technical sense. It may also help shape how teachers view their role, responsibility, and confidence in supporting students with diabetes. This interpretation is consistent with intervention studies showing that diabetes education can strengthen teachers’ factual knowledge alongside their self-efficacy and confidence in managing diabetes-related situations at school [9,13].

Training emerged as one of the most consistent correlates of better outcomes in this study. Teachers who had received prior diabetes-related training reported more favorable attitudes, greater emergency readiness, and better routine practices. This is highly consistent with the available evidence. In Jordan, Allefdawi et al. [9] showed that a brief, structured educational intervention significantly improved teachers’ knowledge, attitudes, and self-efficacy for managing type 1 diabetes in school settings. In northern Saudi Arabia, Alshammari and Haridi [20] found that prior training was a significant predictor of good teacher knowledge. These converging findings strengthen the case that training is not merely desirable but central to improving school preparedness. However, the modest variance explained for emergency readiness (R^2^ = 0.142) and routine practices (R^2^ = 0.140) indicates that teacher cognitions account for only part of these outcomes. Unmeasured organizational factors, such as written school health policies, nurse workload, availability of trained backup staff, administrative support, and access to diabetes supplies, are likely to play an important role alongside knowledge and attitudes.

The finding that school nurse availability was strongly associated with greater emergency readiness is also important. Although only a small proportion of respondents reported access to a school nurse, those who did had better readiness scores. This mirrors the conclusions of Stefanowicz-Bielska et al. [12], who argued that the quality of care for children with type 1 diabetes in schools should be improved through stronger nurse support, ongoing teacher training, and detailed care plans adapted to school conditions. The current study therefore adds to a growing body of evidence suggesting that teacher goodwill cannot substitute for school health infrastructure.

The routine practice findings are similarly telling. Teachers generally endorsed allowing students to monitor blood glucose or eat snacks when needed, and communication with parents or school nurses was strongly supported. However, fewer respondents reported that their schools provided appropriate food options, adapted physical activity where necessary, or organized educational sessions about diabetes. This suggests that schools may be more comfortable accommodating isolated immediate needs than embedding diabetes support into everyday school routines. That pattern is again in line with prior studies showing that schools often lack formalized systems for managing diabetes despite general teacher willingness to help. Previous findings [17,21] point to this broader issue of incomplete institutional preparedness. At a system level, these findings indicate that teacher training should be accompanied by low-resource school protocols rather than depending only on the presence of a school nurse [7,8]. A feasible minimum package would include an individualized diabetes care plan for each student, a written hypoglycemia and emergency algorithm, identification of at least two trained staff members, accessible glucose sources and prescribed glucagon, arrangements for appropriate snacks or food choices, and clear procedures for glucose monitoring, physical activity, parent contact, and escalation to health services [7,8]. Developing these protocols with teachers, school leaders, parents, students, nurses or primary-care teams, and education and health authorities may improve feasibility and shared ownership [7,8].

Several covariate findings are also noteworthy, although they should be interpreted with caution. Teachers who reported having students with diabetes in their classroom had higher knowledge scores, which is in keeping with earlier work suggesting that direct exposure can improve familiarity and confidence. Alshammari and Haridi [20] similarly found that having or having had a student with type 1 diabetes was associated with better knowledge. The association between family history or personal diabetes experience and more favorable attitudes or practices may reflect the influence of lived experience, which could increase empathy and practical understanding.

A few findings were less intuitive. Postgraduate education was associated with lower knowledge scores, and male sex was associated with higher emergency readiness. These results should be interpreted with care. The postgraduate education finding may reflect unmeasured differences in role, exposure, or subject specialization rather than any true disadvantage associated with higher education. The male sex finding is also not entirely consistent with prior literature. For example, Aljefree et al. [18] found that female teachers were more knowledgeable in some areas, whereas male teachers were more willing to accommodate students with type 1 diabetes and attend support programs. This suggests that sex differences may be context-specific and should not be overinterpreted.

These findings have important implications for school-based diabetes support. While teachers generally expressed positive attitudes toward supporting students with diabetes, this was not accompanied by equally strong emergency readiness or consistently supportive routine practices. Only a minority had received prior diabetes-related training, few reported access to a school nurse, and confidence was notably weaker in relation to more serious situations such as unconsciousness and glucagon administration. These findings suggest that professional development should move beyond general awareness workshops and prioritize simulation-based first-aid training, including recognition of severe hypoglycemia, response to an unconscious student, supervised practice with glucagon devices, and rehearsal of school emergency protocols [13]. Overall, this suggests that teacher willingness alone may not be enough to ensure safe and consistent support during the school day. Instead, the findings point to the importance of more structured preparation, including training that addresses both misconceptions about type 1 diabetes and practical emergency management, supported by clearer school protocols and stronger coordination with parents and school health personnel.

### Strengths, Limitations, and Future Directions

A key strength of this study is its broader coverage across public and private schools and multiple regions of Jordan. The sample exceeded the minimum target, the scales showed acceptable to excellent internal consistency, and the analysis extended beyond description by examining how knowledge, attitudes, emergency readiness, and routine practices were interrelated. Another strength is that the study captured both emergency readiness and day-to-day school practices, offering a fuller account of school support than studies focused on knowledge alone. Nevertheless, generalizability to other low- and middle-income countries should be interpreted with caution because school health infrastructure, nurse availability, diabetes-care policies, teacher roles, family-school communication, and cultural expectations regarding chronic disease support may differ substantially across settings.

Furthermore, several limitations should also be noted. First, the cross-sectional design prevents causal interpretation. Although the path model is theoretically plausible and informative, the direction of the observed relationships cannot be established with certainty. Second, the study used convenience sampling and an online survey, which may have introduced selection bias. Teachers with a greater interest in health topics may have been more likely to participate. Therefore, the regional and public/private coverage should be interpreted as sample diversity rather than statistical representativeness. Third, all data were self-reported and may therefore have been influenced by recall bias or social desirability, particularly for items relating to supportive attitudes and school practices. Anonymous online completion was intended to reduce this risk by providing a more private response setting, but residual social desirability bias cannot be excluded [13,14,15]. Fourth, no objective school audit or direct verification was conducted to validate reported emergency preparedness or routine school practices. Specifically, the study did not independently verify whether schools had glucometers, written diabetes protocols, trained staff, or other emergency supplies in place. In addition, the model did not include detailed organizational variables such as school health policy content, nurse workload, staff-to-student coverage, leadership support, or supply availability, which may explain additional variation in emergency readiness and routine practices. Fifth, the lower knowledge score observed among postgraduate participants should be interpreted cautiously. Although professional title was included in the adjusted model, the study did not collect detailed information on teaching subject, postgraduate specialization, administrative role complexity, or prior exposure to student health issues; residual confounding by these factors may partly explain the observed postgraduate education finding. Sixth, prior diabetes-related training was measured only as a yes/no variable; the survey did not capture training duration, recency, accreditation status, content, or whether hands-on emergency practice was included. Therefore, the present data cannot assess a dose–response relationship between training exposure and preparedness. Finally, although the instrument showed good reliability and content review, and underwent pilot testing, and internal consistency assessment, formal construct validation such as exploratory or confirmatory factor analysis was not conducted. The knowledge scale should therefore be interpreted as assessing diabetes-related school-care knowledge in the context of type 1 diabetes, rather than as a purely type 1 diabetes-specific biomedical construct, because some items intentionally covered broader management concepts that remain relevant to school support for children with type 1 diabetes. Future studies should further evaluate the factor structure and measurement properties of the instrument in independent samples.

Future research could extend the current findings by evaluating which types of training are most effective, sustainable, and scalable in school settings. Such studies should record the intensity, recency, accreditation status, and practical components of training so that dose–response effects can be examined and the evidence base for periodic continuing education can be strengthened. The recent Jordanian intervention study [9] offers a useful starting point, but further work is needed in larger and more diverse school contexts. Mixed-methods studies could also explore how teachers, parents, students, and school leaders understand responsibility for diabetes care and where the main practical barriers lie. Observational and audit-based studies would be particularly useful for documenting existing support systems within schools, rather than relying entirely on staff reports.

## 5. Conclusions

Jordanian schoolteachers generally reported positive attitudes toward supporting students with diabetes, and many demonstrated a reasonable level of basic knowledge. However, these strengths were not matched by consistently strong emergency preparedness or by uniformly supportive routine school practices. Knowledge was associated with more favorable attitudes, and attitudes were in turn linked to both emergency readiness and routine practices, while prior diabetes-related training emerged as one of the clearest correlates of better outcomes. Overall, the findings suggest that improving school support for students with diabetes requires more than goodwill. Targeted teacher training, particularly simulation-based first-aid training for severe hypoglycemia and glucagon use, written school protocols, designated trained staff, and stronger school health infrastructure are needed to ensure that students with diabetes can participate safely and fully in school life. These measures should be implemented as system-level school policies, with explicit roles for teachers, administrators, parents, students, and health professionals, rather than left to individual teacher discretion.

## Figures and Tables

**Table 1:** Demographic and school-related characteristics of participating teachers.

Variable		Median (IQR)	Frequency (%)
Age		43 (38–47)	
Marital status	Not married		89 (14.8%)
	Married		512 (85.2%)
Educational level	Diploma		38 (6.3%)
	Bachelor’s degree		458 (75.7%)
	Postgraduate degree		109 (18.0%)
Years of experience		16 (9–22)	
Professional title	Manager		76 (12.7%)
	Teacher		521 (87.3%)
Type of school	Girls		147 (24.4%)
	Boys		85 (14.1%)
	Mixed		370 (61.5%)
Educational sector	Public		485 (80.3%)
	Private		119 (19.7%)
Teaching grades	Elementary		323 (54.5%)
	Secondary		102 (17.2%)
	High school		168 (28.3%)
Gender	Female		535 (88.6%)
	Male		69 (11.4%)
Is there a nurse in your school?	No		530 (87.7%)
	Yes		74 (12.3%)
Have you ever received training in diabetes management?	No		508 (84.1%)
	Yes		96 (15.9%)
Do you have any students with diabetes in your class?	No		340 (56.2%)
	I don’t know		81 (13.4%)
	Yes		184 (30.4%)
Do you have diabetes yourself?	No		563 (93.1%)
	Yes		42 (6.9%)
Do you have a first-degree relative with diabetes?	No		228 (37.8%)
	Yes		375 (62.2%)

**Table 2:** Teachers’ knowledge regarding type 1 diabetes.

Item	Agree	I Don’t Know	Disagree
Type 1 diabetes is a long-term (chronic) condition that requires ongoing management.	518 (85.8%)	62 (10.3%)	24 (4%)
Type 1 diabetes occurs mainly because the pancreas does not produce enough insulin	476 (78.7%)	71 (11.7%)	58 (9.6%)
Eating too much sugar and not exercising are the main causes of Type 1 diabetes *	382 (63%)	63 (10.4%)	161 (26.6%)
Type 1 diabetes can be spread from one person to another. *	113 (18.7%)	123 (20.4%)	367 (60.9%)
Children with Type 1 diabetes can attend school just like other children.	520 (86%)	33 (5.5%)	52 (8.6%)
Children with diabetes can safely take part in physical education and sports activities.	355 (59%)	91 (15.1%)	156 (25.9%)
Regular monitoring of blood glucose levels is essential for children with Type 1 diabetes.	513 (84.8%)	40 (6.6%)	52 (8.6%)
Diabetes may lead to complications such as kidney disease, nerve damage, or vision problems.	491 (81.2%)	59 (9.8%)	55 (9.1%)
Shaking and sweating are common warning signs of low blood sugar (hypoglycemia).	479 (79.2%)	79 (13.1%)	47 (7.8%)
Poorly controlled diabetes can result in weight loss and tiredness.	475 (78.9%)	65 (10.8%)	62 (10.3%)
Insulin therapy is the main treatment for children with Type 1 diabetes.	390 (64.5%)	122 (20.2%)	93 (15.4%)
Insulin use does not cause dependency or addiction.	295 (49.1%)	177 (29.5%)	129 (21.5%)
Maintaining a healthy diet and regular exercise can help prevent episodes of high blood sugar.	512 (84.9%)	35 (5.8%)	56 (9.3%)
Having a family history of diabetes increases a child’s risk of developing the condition.	489 (81%)	55 (9.1%)	60 (9.9%)

Note. Correct response; n (%) indicates the response scored as correct. Correct responses were coded as 1 and all other responses as 0; the 14 items shown in the table were summed to form the knowledge score (range: 0–14). * Indicate that disagree is the correct answer; for the remaining items, agree was the correct answer.

**Table 3:** Teachers’ attitudes toward students with diabetes.

Item	Strongly Disagree	Disagree	Neutral	Agree	Strongly Agree
I am willing to deal with a student with diabetes in my class.	15 (2.5%)	39 (6.5%)	120 (19.9%)	205 (34.0%)	224 (37.1%)
I would like to attend training programs on managing students with diabetes.	3 (0.5%)	28 (4.6%)	85 (14.1%)	226 (37.4%)	262 (43.4%)
Students with diabetes should be treated like all other students in school.	11 (1.8%)	61 (10.1%)	66 (11.0%)	206 (34.2%)	258 (42.9%)
I worry about the safety of students with diabetes during school hours.	6 (1.0%)	19 (3.2%)	58 (9.6%)	251 (41.6%)	269 (44.6%)
Teachers should be trained to handle hypoglycemia and hyperglycemia in school.	7 (1.2%)	17 (2.8%)	43 (7.1%)	188 (31.2%)	347 (57.6%)
Having a school nurse is essential to ensure the safety of students with diabetes.	4 (0.7%)	12 (2.0%)	29 (4.8%)	138 (23.0%)	418 (69.6%)
Schools should have a glucometer available for emergency use.	6 (1.0%)	8 (1.3%)	30 (5.0%)	147 (24.5%)	409 (68.2%)
Educational programs about diabetes should be mandatory for all teachers.	3 (0.5%)	39 (6.5%)	80 (13.3%)	188 (31.2%)	310 (51.5%)
I believe students with diabetes can perform well academically if supported.	6 (1.0%)	28 (4.6%)	120 (19.9%)	149 (24.8%)	405 (67.3%)
I believe students with diabetes are a burden on the school and teachers.	116 (19.2%)	122 (20.2%)	106 (17.6%)	169 (28.0%)	90 (14.9%)

**Table 4:** Teachers’ confidence and emergency preparedness for managing students with diabetes.

	Strongly Disagree	Disagree	Neutral	Agree	Strongly Agree
I am confident in my ability to recognize signs of hypoglycemia (such as sweating, trembling, or confusion).	17 (2.8%)	64 (10.6%)	152 (25.2%)	193 (32.1%)	176 (29.2%)
I know the correct first step to take if a student with diabetes reports feeling weak.	21 (3.5%)	84 (14%)	155 (25.8%)	209 (34.8%)	132 (22%)
I know the appropriate actions to take if a student with diabetes becomes unconscious.	33 (5.5%)	109 (18.2%)	180 (30.1%)	161 (26.9%)	116 (19.4%)
My school has a glucometer available, and staff members (including myself) know how to use it in an emergency.	71 (11.8%)	154 (25.6%)	126 (21%)	132 (22%)	118 (19.6%)
My school has at least one designated staff member trained to manage diabetic emergencies.	62 (10.3%)	113 (18.8%)	148 (24.7%)	146 (24.3%)	131 (21.8%)
I know how to administer glucagon if a student with diabetes loses consciousness.	102 (17%)	155 (25.8%)	134 (22.3%)	120 (20%)	89 (14.8%)
My school has a written emergency plan or protocol specifically for students with diabetes.	68 (11.4%)	119 (19.9%)	163 (27.3%)	124 (20.8%)	123 (20.6%)

**Table 5:** Routine school practices related to diabetes management.

Item	Strongly Disagree	Disagree	Neutral	Agree	Strongly Agree
Students with diabetes are allowed to eat snacks or check their blood glucose during class when needed.	9 (1.5%)	33 (5.5%)	128 (21.3%)	198 (33%)	232 (38.7%)
Teachers should communicate regularly with parents or the school nurse about the needs of students with diabetes.	9 (1.5%)	17 (2.8%)	77 (12.8%)	204 (33.9%)	294 (48.9%)
My school provides food options suitable for students with diabetes.	54 (9%)	100 (16.6%)	163 (27.1%)	155 (25.8%)	129 (21.5%)
At our school, physical activities are adjusted or modified for students with diabetes when necessary.	34 (5.7%)	89 (14.8%)	176 (29.3%)	161 (26.8%)	140 (23.3%)
My school organizes educational sessions or awareness activities about diabetes.	32 (5.3%)	75 (12.5%)	152 (25.3%)	200 (33.3%)	142 (23.6%)
Teachers should encourage students to adopt healthy lifestyle habits.	8 (1.3%)	19 (3.2%)	71 (11.8%)	158 (26.3%)	345 (57.4%)

**Table 6:** Direct effects from the final path model (including demographic and school covariates).

Outcome	Predictor	b	SE	95% CI	β	*p*
Knowledge (K)	Age (years)	0.047	0.024	[0.004, 0.097]	0.126	0.046
	Years of experience	−0.046	0.021	[−0.089, −0.003]	−0.138	0.030
	Male (ref: Female)	−0.722	0.699	[−2.053, 0.681]	−0.08	0.302
	Married (ref: Not married)	0.503	0.369	[−0.243, 1.240]	0.062	0.173
	Diploma (ref: Bachelor’s)	0.246	0.358	[−0.533, 0.972]	0.021	0.493
	Postgraduate (ref: Bachelor’s)	−0.826	0.373	[−1.645, −0.134]	−0.11	0.027
	Manager (ref: Teacher)	−0.267	0.37	[−1.016, 0.456]	−0.031	0.470
	Female school (ref: Mixed)	0.401	0.296	[−0.235, 1.060]	0.06	0.175
	Male school (ref: Mixed)	0.309	0.619	[−0.950, 1.332]	0.038	0.617
	Private sector (ref: Public)	−0.324	0.331	[−1.043, 0.261]	−0.045	0.328
	Secondary grades (ref: Elementary)	−0.54	0.312	[−1.294, −0.010]	−0.071	0.084
	High-school grades (ref: Elementary)	0.09	0.291	[−0.545, 0.633]	0.014	0.758
	Nurse available: Yes (ref: No)	0.254	0.373	[−0.541, 0.957]	0.029	0.496
	Nurse available: Unknown (ref: No)	−1.498	0.673	[−2.669, −0.353]	−0.086	0.026
	Diabetes training: Yes (ref: No)	0.575	0.336	[−0.154, 1.265]	0.073	0.087
	Students with diabetes: Don’t know (ref: No)	0.267	0.384	[−0.550, 1.066]	0.032	0.486
	Students with diabetes: Yes (ref: No)	0.967	0.229	[0.541, 1.417]	0.154	<0.001
	Respondent has diabetes: Yes (ref: No)	0.148	0.473	[−0.900, 1.002]	0.013	0.754
	Family history (1st-degree): Yes (ref: No)	0.287	0.22	[−0.204, 0.697]	0.048	0.192
	Family history (1st-degree): Unknown (ref: No)	−1.338	0.799	[−2.979, 0.370]	−0.07	0.094
Attitude (A)	Knowledge (K)	0.699	0.098	[0.494, 0.904]	0.374	<0.001
	Age (years)	0.001	0.045	[−0.106, 0.087]	0.001	0.986
	Years of experience	−0.051	0.037	[−0.121, 0.013]	−0.082	0.164
	Male (ref: Female)	−0.972	1.299	[−3.980, 1.170]	−0.057	0.455
	Married (ref: Not married)	−0.972	0.618	[−2.116, 0.234]	−0.064	0.116
	Diploma (ref: Bachelor’s)	−0.114	0.913	[−2.261, 1.775]	−0.005	0.901
	Postgraduate (ref: Bachelor’s)	0.303	0.563	[−0.819, 1.518]	0.022	0.590
	Manager (ref: Teacher)	−1.337	0.682	[−2.737, −0.022]	−0.083	0.050
	Female school (ref: Mixed)	0.666	0.492	[−0.254, 1.658]	0.053	0.176
	Male school (ref: Mixed)	−0.106	1.159	[−2.246, 2.570]	−0.007	0.927
	Private sector (ref: Public)	0.982	0.551	[−0.135, 2.042]	0.073	0.075
	Secondary grades (ref: Elementary)	0.122	0.49	[−0.866, 1.060]	0.009	0.803
	High-school grades (ref: Elementary)	−0.942	0.5	[−1.871, 0.018]	−0.079	0.060
	Nurse available: Yes (ref: No)	0.724	0.534	[−0.310, 1.731]	0.044	0.175
	Nurse available: Unknown (ref: No)	0.042	1.024	[−1.888, 2.072]	0.001	0.967
	Diabetes training: Yes (ref: No)	1.195	0.471	[0.182, 2.139]	0.081	0.011
	Students with diabetes: Don’t know (ref: No)	−0.27	0.598	[−1.478, 0.978]	−0.017	0.652
	Students with diabetes: Yes (ref: No)	0.025	0.477	[−0.935, 1.119]	0.002	0.959
	Respondent has diabetes: Yes (ref: No)	1.979	0.714	[0.539, 3.395]	0.094	0.006
	Family history (1st-degree): Yes (ref: No)	1.003	0.46	[−0.049, 1.778]	0.09	0.029
	Family history (1st-degree): Unknown (ref: No)	1.877	1.043	[−0.193, 4.001]	0.053	0.072
Emergency readiness (C)	Attitude (A)	0.178	0.053	[0.070, 0.290]	0.141	<0.001
	Age (years)	−0.052	0.052	[−0.158, 0.064]	−0.059	0.318
	Years of experience	−0.037	0.046	[−0.124, 0.060]	−0.047	0.423
	Male (ref: Female)	3.008	1.467	[0.042, 6.134]	0.14	0.040
	Married (ref: Not married)	1.418	0.799	[−0.185, 3.289]	0.074	0.076
	Diploma (ref: Bachelor’s)	0.988	1.038	[−0.800, 3.145]	0.035	0.341
	Postgraduate (ref: Bachelor’s)	0.421	0.685	[−1.006, 1.833]	0.024	0.539
	Manager (ref: Teacher)	2.106	0.748	[0.723, 3.865]	0.103	0.005
	Female school (ref: Mixed)	1.038	0.669	[−0.297, 2.398]	0.066	0.121
	Male school (ref: Mixed)	−0.727	1.406	[−3.752, 2.055]	−0.038	0.605
	Private sector (ref: Public)	0.935	0.724	[−0.340, 2.540]	0.055	0.196
	Secondary grades (ref: Elementary)	−0.588	0.755	[−2.229, 0.909]	−0.033	0.436
	High-school grades (ref: Elementary)	−0.72	0.635	[−1.981, 0.495]	−0.048	0.256
	Nurse available: Yes (ref: No)	3.042	0.775	[1.755, 4.578]	0.147	<0.001
	Nurse available: Unknown (ref: No)	1.764	1.201	[−0.391, 4.229]	0.043	0.142
	Diabetes training: Yes (ref: No)	3.268	0.707	[1.825, 4.728]	0.176	<0.001
	Students with diabetes: Don’t know (ref: No)	0.616	0.788	[−0.972, 2.219]	0.031	0.435
	Students with diabetes: Yes (ref: No)	0.599	0.64	[−0.806, 1.867]	0.041	0.349
	Respondent has diabetes: Yes (ref: No)	1.506	1.007	[−0.457, 3.694]	0.056	0.135
	Family history (1st-degree): Yes (ref: No)	0.899	0.644	[−0.432, 2.022]	0.064	0.163
	Family history (1st-degree): Unknown (ref: No)	−3.304	1.409	[−5.925, −0.400]	−0.073	0.019
Routine practices (S)	Attitude (A)	0.204	0.034	[0.143, 0.282]	0.244	<0.001
	Knowledge (K)	0.127	0.055	[0.018, 0.232]	0.082	0.021
	Age (years)	0.011	0.036	[−0.055, 0.097]	0.019	0.760
	Years of experience	−0.015	0.031	[−0.080, 0.053]	−0.029	0.638
	Male (ref: Female)	0.911	0.981	[−1.030, 3.033]	0.065	0.353
	Married (ref: Not married)	−0.035	0.557	[−1.190, 1.035]	−0.003	0.950
	Diploma (ref: Bachelor’s)	0.666	0.813	[−0.796, 2.370]	0.036	0.413
	Postgraduate (ref: Bachelor’s)	−0.186	0.48	[−1.109, 0.725]	−0.016	0.698
	Manager (ref: Teacher)	0.774	0.544	[−0.410, 1.850]	0.058	0.155
	Female school (ref: Mixed)	−0.019	0.447	[−0.857, 0.816]	−0.002	0.966
	Male school (ref: Mixed)	−0.758	0.988	[−2.803, 1.161]	−0.06	0.443
	Private sector (ref: Public)	0.202	0.467	[−0.609, 1.127]	0.018	0.666
	Secondary grades (ref: Elementary)	−0.194	0.448	[−1.045, 0.716]	−0.016	0.664
	High-school grades (ref: Elementary)	0.011	0.392	[−0.763, 0.702]	0.001	0.977
	Nurse available: Yes (ref: No)	0.593	0.653	[−0.683, 1.891]	0.043	0.364
	Nurse available: Unknown (ref: No)	−0.573	0.81	[−2.133, 1.029]	−0.021	0.479
	Diabetes training: Yes (ref: No)	1.294	0.487	[0.192, 2.383]	0.106	0.008
	Students with diabetes: Don’t know (ref: No)	−0.26	0.516	[−1.360, 0.889]	−0.02	0.615
	Students with diabetes: Yes (ref: No)	−0.143	0.392	[−1.024, 0.688]	−0.015	0.715
	Respondent has diabetes: Yes (ref: No)	0.429	0.664	[−0.915, 1.799]	0.024	0.518
	Family history (1st-degree): Yes (ref: No)	0.842	0.371	[0.025, 1.546]	0.091	0.023
	Family history (1st-degree): Unknown (ref: No)	−1.88	1	[−3.789, 0.112]	−0.063	0.060

Note. b = unstandardized coefficient; SE = standard error; 95% CI = percentile bootstrap confidence interval; β = standardized coefficient. Dummy variables were coded as 1 = category and 0 = reference. Reference categories were female, not married, bachelor’s degree, teacher, mixed school, public sector, elementary grades, no nurse, no training, no students with diabetes, respondent without diabetes, and no first-degree family history of diabetes. Region was excluded from the model. The direct path from knowledge to emergency readiness was constrained to zero.

**Table 7:** Indirect and total effects from the final path model.

Effect	b	SE	95% CI	Β	*p*
Knowledge → Emergency readiness (indirect via Attitude)	0.125	0.041	[0.044, 0.203]	0.05273232	0.053
Knowledge → Emergency readiness (total)	0.125	0.041	[0.044, 0.203]	0.05273232	0.053
Knowledge → Routine practices (indirect via Attitude)	0.142	0.031	[0.088, 0.201]	0.09145748	0.091
Knowledge → Routine practices (total)	0.269	0.054	[0.162, 0.372]	0.17298200	0.173

Note: b = unstandardized effect; SE = standard error; 95% CI = percentile bootstrap confidence interval; β = standardized effect. Total effect for Knowledge → Emergency readiness equals the indirect effect because the direct path was constrained.

## Data Availability

The data presented in this study are available on request from the corresponding author due to participants’ privacy.

## Abbreviations

The following abbreviations are used in this manuscript:

CFI	comparative fit index
CI	confidence interval
IQR	interquartile range
MLR	maximum likelihood estimation with robust standard errors
R^2^	coefficient of determination
RMSEA	root mean square error of approximation
SRMR	standardized root mean square residual
T1DM	type 1 diabetes mellitus
VIF	variance inflation factor

## Appendix A

**Table A1:** Missingness frequency for variables included in the final adjusted path model.

Variable	Non-Missing n	Missing n (%)
Knowledge score	606	5 (0.8%)
Attitude score	603	8 (1.3%)
Emergency readiness score	600	11 (1.8%)
Routine practices score	601	10 (1.6%)
Age	591	20 (3.3%)
Teaching experience	570	41 (6.7%)
Sex	604	7 (1.1%)
Marital status	601	10 (1.6%)
Educational level	605	6 (1.0%)
Professional title	597	14 (2.3%)
School type	602	9 (1.5%)
School sector	604	7 (1.1%)
Grade level taught	593	18 (2.9%)
School nurse availability	604	7 (1.1%)
Prior diabetes-related training	604	7 (1.1%)
Students with diabetes in classroom	605	6 (1.0%)
Respondent diabetes status	605	6 (1.0%)
First-degree family history of diabetes	603	8 (1.3%)

Note: The analysis dataset contained 611 records. Missingness was evaluated for the variables included in the final adjusted path model. Complete-case refitting used 519 participants with complete data across all listed variables.
